# Supplementary material for: Healthcare Access in the Aftermath: A Longitudinal Analysis of Disaster Impact on US Communities
Source: Int J Environ Res Public Health. 2025 May 5;22(5):733. doi: 10.3390/ijerph22050733 (PMC12111409; doi:10.3390/ijerph22050733)
Supplement: Supplementary file 1 [file ijerph-22-00733-s001.zip › ijerph-3527314-supplementary.pdf]

**Table S1.** National Establishment Time Series (NETS) Data of Healthcare Facilities					
**Domain**	**3 Letter Main Category NET Code**	**Definition**	**3 letter Subcategory NET Code**	**8 -Digit SIC Code**	**Subcategory Description**
Pharmacies and drug stores	DRA	Pharmacies and drug stores (where medicine and/or drugs are dispensed or sold) —> drug stores and pharmacies. Locations representing stores where medicine and/or drugs are dispensed or sold (may not include those that are also supermarkets).	DRG	59120000-59129999, 80110203	of pharmacies and drug stores – SIC code-based definition
			DRN	53000000-53999999, 54000000-54999999, 56000000-56999999, 59000000-59999999, 65120200-65120299	of pharmacies and drug stores – chain name-based definition
Hospital based inpatient care	HOI	All hospitals are major medical centers	MHH	80630000, 80639900, 80639901	Mental Health Hospitals
			BHH	80690100, 80690101, 80690102	Behavioral Health Hospitals
			HOS	80620000-80629999, 80110200, 80110201, 80690000, 80690200, 80690201, 80690300, 80690301, 80699900, 80699902, 80699903, 80699904, 80699905	Hospitals or major medical centers
Ambulatory Care	AMB	Locations able to treat and provide chronic, continuous, outpatient care including screenings and other preventive measures. Places both allow an individual to maintain their health and address non-acute,	MHO	80110400, 80110401, 80110402, 80110403, 80490400, 80490401, 80490403, 80490404, 80939902	Mental health outpatient and continuous care
			BHO	80930100, 80930101,	Behavior health outpatient and continuous care

		non-emergency conditions.		80930102, 80930103	
			URG	80110204	Urgent Care
			RTC	80990103, 80990201	Retail clinics
			PHT	80110521, 80490200, 80490201, 80939903	Physical therapists
			KCT	80920000	Kidney centers
			HPC	80110100-80110199, 80110500-80110514, 80119900-80119999, 80490100-80490199, 80930200-80930299, 80930300-80930399, 80110000, 80110202, 80110205, 80110517, 80110518, 80110519, 80110520, 80110522, 80110523, 80110524, 80310000, 80410000, 80420000, 80420100, 80420101, 80420102, 80420103, 80420105, 80429900, 80429901, 80430000, 80490000, 80499900, 80499902, 80499904, 80499906, 80499908, 80499909, 80930000, 80939900, 80939905, 80990200,	Offices or clinics of health practitioners

				80999906, 80999907	
			DDS	80210100-80210105, 80210000, 80210107, 80210108, 80210200, 80210201, 80210202, 80219902	Dental Care

Table S2. Sensitivity Analysis of Adjusted Autoregressive Models: Change in Total Number of Healthcare Establishments in 2014 With Total Number of Moderate and Severe Climate-related Disasters for 3108 Nonwater US Counties

	**Pharmacies**			**Hospitals**			**Ambulatory Care**		
	**Count (β)**	**SE**	**95 % CI**	**Count (β)**	**SE**	**95 % CI**	**Count (β)**	**SE**	**95 % CI**
Climate-related Disasters									
Moderate Disaster	0.09	0.08	(-0.06, 0.24)	0.14*	0.06	(0.03, 0.25)	2.52**	0.82	(0.91, 4.12)
Severe Disaster	0.10	0.11	(-0.12, 0.32)	-0.31***	0.08	(-0.47, -0.14)	-5.99***	1.20	(-8.35, -3.64)
Model Fit	R^2=0.85			R^2=0.89			R^2=0.93		
N=	3108								

* $p < 0.05$; ** $p < 0.01$; *** $p < 0.001$. Counts were adjusted for poverty rates in 2000, the total number of healthcare establishments in 2000, total population in 2014, and non-Hispanic white residents.
